# Protein sources and starch-protein digestive dynamics manipulate growth performance in broiler chickens defined by an equilateral-triangle response surface design

**DOI:** 10.1016/j.aninu.2022.01.003

**Published:** 2022-02-03

**Authors:** Shemil P. Macelline, Peter V. Chrystal, Peter H. Selle, Sonia Y. Liu

**Affiliations:** a Poultry Research Foundation, The University of Sydney, Camden NSW 2570, Australia; b School of Life and Environmental Sciences, Faculty of Science, The University of Sydney, Sydney NSW 2006, Australia; c Sydney School of Veterinary Science, The University of Sydney, Sydney NSW 2006, Australia

**Keywords:** Amino acid, Digestive dynamics, Glucose, Protein, Starch

## Abstract

A total of 360 male, off-sex Ross 308 chicks were offered 10 dietary treatments from 14 to 35 d post–hatch in an equilateral-triangle response surface design feeding study in order to confirm the importance of protein and amino acid digestive dynamics in broiler chickens. The 3 apical diets were nutritionally-equivalent containing either soybean meal, non-bound amino acids or whey protein concentrate as the major source of dietary protein and amino acids. Appropriate blends of the 3 apical diets comprised the balance of 7 diets and each dietary treatment was offered to 6 replicate cages with 6 birds per cage. Growth performance, nutrient utilisation, apparent protein and starch digestibility coefficients were determined in 4 small intestinal segments. The optimal weight gain (2,085 g/bird) and feed conversion ratios (FCR, 1.397) were generated by Diet 50S50W which included a 50:50 blend of apical diets rich in whey protein concentrate and soybean meal. Broiler chickens offered Diet 50S50W also had the highest experimental and predicted jejunal digestibility (0.685 in proximal jejunum and 0.823 in distal jejunum). FCR was not correlated with apparent distal ileal digestibility coefficient (*P* > 0.05) of protein but was correlated with apparent protein digestibility in proximal jejunum (*r =* −0.369, *P* = 0.040) and distal jejunum (*r =* −0.316, *P* = 0.015). Surplus dietary starch was correlated with increased fat pad weight (*r =* 0.781, *P* = 0.008). The findings confirmed the relevance of protein digestion rate, reflected by jejunal digestibility, on feed conversion of broiler chickens. A balance between protein-bound and non-bound crystalline or synthetic amino acids may be required for optimal growth and protein digestion.

## 1 Introduction

The digestive dynamics of protein and starch have been shown to influence broiler growth performance, especially in diets containing high inclusion levels of non-bound crystalline or synthetic amino acids (NBAA) (Liu and Selle, 2015; Selle and Liu, 2019). The implication is that a precise balance between intestinal uptakes of glucose and amino acids will advantage protein deposition and ultimately growth performance. Digestibility coefficients determined at the end of small intestine represent the extent of nutrient digestion which is extremely important for growth performance; however, the rate and site of nutrient digestion may also be crucial considering jejunum is the major site of glucose and amino acid absorption (Riesenfeld et al., 1980). Sydenham et al. (2017) reported quadratic relationships between jejunal starch:protein disappearance rate ratios and both weight gains and feed conversion ratios (FCR) in broiler chickens, which suggested a balance between glucose and amino acid uptakes is needed for optimal growth performance. Instructively, Moughan (2003) concluded that efficient protein synthesis was dependent upon the harmonious availability of amino acids and non-amino acid energy sources at sites of protein synthesis. The energy cost of whole-body protein synthesis was calculated to be 5.35 kJ per g protein synthesis, and hence on a molar basis 7.52 adenosine triphosphates (ATP) are required per peptide bond synthesis (Aoyagi et al., 1988). Moreover, an appropriate balance of amino acids is required and amino acid imbalance may depress growth performance by potentially inhibiting feed intake and/or retarding the rate of protein deposition (Melnick et al., 1946; Gupta et al., 1958).

Liu and Selle (2015) reported that in a series of studies, feed conversion was improved with either rapidly digestible protein or slowly digestible starch; however, the impact of protein digestion rate on FCR was greater than the rate of starch digestion. Moreover, soybean meal is the primary source of protein in typical poultry diets, and strategies to reduce the inclusion of soybean meal require utilisation of alternative protein sources including higher inclusions of NBAA (Chrystal et al., 2020a, Chrystal et al., 2020b). Moss et al. (2018) reported that proximal ileal starch digestibility coefficients were negatively correlated with digestibility coefficients of 12 amino acids in broiler chickens. Thus, evaluations of different protein sources should not be considered in isolation from the starch component of the diet. This raises the possibility that glucose and amino acids were competing for intestinal uptakes via their respective Na^+^-dependent transport systems. Moreover, Gupta et al. (1958) compared protein digestibility in the stomach and small intestine of rats offered diets containing 150 g/kg of casein, zein, beef proteins or an amino acid mixture and they reported that the digestion of zein was slower than that of the other 3 sources of protein. Recently, Truong et al. (2017) tested the hypothesis that different protein source will not only lead to variations in the extent of protein digestion but also rate of digestion. This was confirmed by significantly higher jejunal protein digestibility observed in broiler chickens offered the diet containing highest level of casein and lowest level of soybean meal.

In the present study, the protein sources chosen (NBAA; whey protein concentrate, WPC; and soybean meal, SBM) represented fast, medium, and slow rates of digestion, respectively. Three iso-energetic wheat-based diets were formulated to contain similar true protein content but different inclusions of soybean meal (71%), NBAA (31%) and whey protein (56%) where their approximate percentage contributions to dietary protein are shown in parentheses. The balance of 7 dietary treatments was comprised of appropriate blends of the 3 apical diets. Whey protein is not incorporated into practical broiler diets, but it is considered as a more rapid digestible source of protein than soybean meal (Dangin et al., 2001). In poultry, Pineda-Quiroga et al. (2018) reported that inclusions of both dry whey powder (60 g/kg) or WPC (80 g/kg) in broiler diets enhanced growth performance and NBAA do not require digestion and are rapidly absorbed along the small intestine (Wu, 2009). Following Truong et al. (2017), the primary goal of the present study is to confirm the importance of starch and protein, protein-bound amino acids, and NBAA digestive balance in broiler chickens by visualising the optimal dietary composition on growth performance and nutrient utilisations via an equilateral-triangle response surface design.

## 2 Materials and methods

### 2.1 Animal ethics

All experimental procedures were specifically approved by the Animal Research Authority of the University of Sydney (Project number 2019/1516).

### 2.2 Experimental design and diet preparation

An equilateral triangle response surface design was applied in the present study. Three iso-energetic (12.9 MJ/kg ME) and iso-nitrogenous (203 g/kg true protein) wheat-based apical diets were formulated to provide different sources of protein and amino acids. Three apical diets (100S, 100A, and 100W) were formulated based on near-infrared spectroscopy (NIR) specifications of wheat and soybean meal using the AMINONir Advanced programme (Evonik Nutrition & Care Gmbh, Hanau, Germany). Diet 100S was based on soybean meal with low inclusions of NBAA (6.75 g/kg). Diet 100A contained the highest level of NBAA (66.9 g/kg). Diet 100W included whey protein concentrate (Fonterra Australia Pty Ltd. Vic, Australia) and moderate level of NBAA (19 g/kg) as a total replacement for soybean meal. The remaining 7 experimental diets were derived from blending the 3 apical diets based on ratios shown in Table 1. The 3 apical diets contained identical levels of digestible lysine (11.5 g/kg) and similar ideal protein ratios for essential amino acids. Dietary composition and nutrient specifications of the 3 apical diets are shown in Table 2, Table 3. A dietary marker (Celite World Minerals, Lompoc, CA, USA) was included at 20 g/kg as an inert acid insoluble ash (AIA) marker in order to determine starch and protein (N) digestibility coefficients in 4 small intestinal sites. All diets were steam-pelleted at 80 °C and then offered to broiler chickens from 14 to 35 d post–hatch. A commercial starter diet based on wheat and soybean meal with 12.13 MJ/kg energy and 220 g/kg crude protein (CP), was offered to broiler chickens from 1 to 13 d post–hatch. Pellet durability index (PDI) of all diets was determined using the NHP 200 New Holmen Automatic Pellet Tester (TekPro Ltd, Norfolk, UK).

**Table 1:** Outline of the 10 dietary treatments based on 3 apical diets (g/100 g) in which protein sources are largely derived from soybean meal (SBM), non-bound amino acids (NBAA) and whey protein concentrate (WPC).

Treatments	SBM	NBAA	WPC	Abbreviations
1	100			100S
2		100		100A
3			100	100W
4	50		50	50S50W
5	50	50		50S50A
6		50	50	50A50W
7	66.6	16.7	16.7	67S17A17W
8	16.7	66.6	16.7	17S67A17W
9	16.7	16.7	66.6	17S17A67W
10	33.3	33.3	33.3	33S33A33W

**Table 2:** Composition of common starter diet and 3 apical diets (as-fed basis, g/kg).

Item	Common starter	100S	100A	100W
Wheat^1^	603	577	689	748
Soybean meal^1^	288	323	162	–
Whey protein^2^	–	–	–	161
Canola meal	70.0	–	–	–
Soybean oil	7.00	47.9	27.5	12.6
Limestone	9.90	11.9	12.4	12.8
Dicalcium phosphate	10.4	6.77	8.35	10.1
Sodium chloride	1.70	3.21	–	–
Sodium bicarbonate	2.40	–	4.71	4.76
Potassium carbonate	–	–	4.40	8.07
L-Lysine HCl	2.10	2.27	7.23	–
DL-Methionine	2.90	3.06	4.52	1.36
L-Threonine	0.90	1.30	3.56	–
L-Tryptophan	–	–	0.22	–
L-Valine	–	0.59	3.45	0.21
L-Isoleucine	–	0.03	2.84	–
L-Leucine	–	–	3.10	–
L-Arginine	–	–	4.10	5.74
L-Histidine	–	–	0.65	0.25
Glycine	–	–	2.39	2.70
L-Serine	–	–	2.91	0.41
L-Glutamic acid	–	–	33.5	8.76
Xylanase	0.10	0.10	0.10	0.10
Phytase	0.10	0.10	0.10	0.10
Choline chloride 60%	–	0.45	1.43	1.45
Vitamin-mineral premix^3^	2.00	2.00	2.00	2.00
Celite	–	20.0	20.0	20.0
Total non-bound amino acids	5.90	6.75	66.9	19.4

1 Near infrared ray analyses values.

2 Whey protein concentrates (800 g/kg crude protein, glutamic acid 137 g/kg; leucine 85 g/kg; aspartic acid 80 g/kg; lysine 73 g/kg; threonine 54 g/kg; proline 47 g/kg; valine 46 g/kg; isoleucine 45 g/kg; alanine 42 g/kg; serine 38 g/kg; phenylalanine 28 g/kg; tyrosine 25 g/kg; arginine 21 g/kg; glycine 17 g/kg; tryptophan 15 g/kg); xylanase (Danisco, Dupont Nutrition & Bioscience, København, Denmark) 40,000 U/g; phytase (Axtra PHY, Dupont Nutrition & Bioscience, København, Denmark) 10,000 FTU.

3 Vitamin-trace mineral premix supplied per tonne of feed retinol 12 million international units (MIU), cholecalciferol 5 MIU, tocopherol 50 g, menadione 3 g, thiamine 3 g, riboflavin 9 g, pyridoxine 5 g, cobalamin 0.025 g, niacin 50 g, pantothenate 18 g, folate 2 g, biotin 0.2 g, copper 20 g, iron 40 g, manganese 110 g, cobalt 0.25 g, iodine 1 g, molybdenum 2 g, zinc 90 g, selenium 0.3 g.

**Table 3:** Calculated nutrient specifications of the 3 apical diets (as-is, g/kg).

Item	100S	100A	100W
AME, MJ/kg	12.9	12.9	12.9
True protein	203	203	203
Crude protein	222	204	214
Starch	387	460	498
Crude fibre	19.3	16.0	12.0
Acid detergent fibre	31.2	25.9	19.5
Neutral detergent fibre	87.3	82.9	73.3
Calcium	8.70	8.70	8.70
Total phosphorous	4.66	4.21	3.70
Phytic phosphorous	2.00	1.55	1.02
Non-phytic phosphorous	2.66	2.66	2.68
Available phosphorus	4.35	4.35	4.35
Digestible amino acids			
Lysine	11.5	11.5	11.5
Methionine	5.7	6.3	4.7
Cysteine	2.8	2.2	4.0
Threonine	7.7	7.7	8.3
Tryptophan	2.5	1.9	2.8
Isoleucine	8.1	8.1	8.6
Leucine	13.8	12.3	16.3
Arginine	12.6	12.0	12.0
Valine	9.2	9.2	9.2
Glycine	7.4	7.4	7.4
Serine	9.1	9.1	9.1
DEB, mEq/kg	242	240	240
Analysed values			
Gross energy, MJ/kg	19.1	18.5	18.7
Crude protein	214	197	208
Starch	325	415	426
Starch:protein ratio	1.53	2.11	2.05

DEB = dietary electrolyte balance.

### 2.3 Bird management

Each of the 10 dietary treatments was offered to 6 replicate cages (6 birds per cage). A total of 360 off-sex male Ross 308-d old chicks (parent line) were procured from a commercial hatchery and offered a common starter diet (Table 2). At 14 d post–hatch, birds were individually identified (wing-tags), weighed and re-allocated into bioassay cages based on body weights so that cage body weight means and variations were effectively identical (528 ± 4.1 g/bird). Thereafter, birds were offered the experimental diets until 35 d post–hatch. Birds had unlimited access to feed and water under a ‘18-h-on-6-h-off’ lighting regime in an environmentally controlled facility. The dimensions of the cages were 750 mm in width and depth and 500 mm in height. An initial room temperature of 32 ± 1 °C was maintained for the first week, which was gradually decreased to 22 ± 1 °C by the end of the third week and maintained at this temperature for the duration of the feeding study. Initial and final body weights were determined, and feed intakes were recorded from which FCR were calculated. The incidence of dead or culled birds was recorded daily and their body-weights used to adjust feed intake and FCR calculations.

### 2.4 Sample collection and chemical analysis

The molecular weight distributions of peptides in wheat, soybean meal and whey protein were assessed by peptide size-exclusion chromatography by methodology similar to that described by Irvine (2003). Amino acid concentrations of 3 apical diets were determined via 24 h liquid hydrolysis at 110 °C in 6 mol/L HCl followed by analysis of 16 amino acids using the Waters AccQTag Ultra chemistry on a Waters Acquity UPLC (Waters Corporation. Milford, Massachusetts). The analysed total amino acid concentrations in the 3 apical diets are shown in Table 4. To determine nutrient utilisation parameters total excreta were collected and weighed from 27 to 29 d post–hatch from trays fitted underneath each cage and feed intakes monitored. The parameters included apparent metabolizable energy (AME), metabolizable energy to gross energy ratios (ME:GE), N retention and N-corrected apparent metabolizable energy (AMEn). Excreta were dried in a forced-air oven at 80 °C for 24 h and the gross energy (GE) of excreta and diets were determined using an adiabatic bomb calorimeter (Parr 1281 bomb calorimeter, Parr Instruments Co., Moline, IL, USA). The AME values of the diets were calculated on a dry matter basis from the following equation:

$${\text{AME}}_{\text{diet}}\left(\text{MJ}/\text{kg}\right)=\frac{\left(\text{Feed intake }\times {\text{ GE}}_{\text{diet}}\right)-\left(\text{Excreta output }\times {\text{ GE}}_{\text{excreta}}\right)}{\text{Feed intake}}$$

**Table 4:** Analysed amino acids composition of 3 apical diets (g/kg).

Item	100S	100A	100W
Arginine	17.7	16.3	14.3
Histidine	7.5	5.9	5.7
Isoleucine	12.8	11.8	14.2
Leucine	21.4	18.1	24.1
Lysine	17.1	16.8	16.7
Methionine	5.3	7.6	5.7
Phenylalanine	14.0	9.5	9.7
Threonine	12.1	11.2	14.5
Valine	14.4	13.7	14.2
Alanine	11.6	7.9	11.7
Aspartic acid	27.2	16.8	22.4
Glutamic acid	59.4	86.5	66.1
Glycine	12.2	11.5	10.6
Proline	17.5	13.8	18.8
Serine	14.0	13.2	13.2
Tyrosine	6.1	4.2	4.3

N contents of diets and excreta were determined using a nitrogen determinator (Leco Corporation, St Joseph, MI) and N retentions calculated from the following equation:

$$\text{Retention }\left(\text{\%}\right)=\frac{\left(\text{Feed intake }\times {\text{ Nutrient}}_{\text{diet}}\right)-\left(\text{Excreta output }\times {\text{ Nutrient}}_{\text{excreta}}\right)}{\text{Feed intake }\times {\text{ Nutrient}}_{\text{diet}}}\times 100$$

N-corrected AME (AMEn MJ/kg DM) values were calculated by correcting N retention to 0 using the factor of 36.54 kJ/g N retained in the body (Hill and Anderson, 1958).

On d 35, birds were euthanised by intravenous injection of sodium pentobarbitone and abdominal fat-pads were dissected, removed, and weighed for each cage. Relative fat-pad weights were then calculated from average body weights in respective cages. Apparent digestibility coefficients and disappearance rates (g/d per bird) of starch and protein were determined in the proximal and distal jejunum and proximal and distal ileum. Digesta samples were collected in their entirety from 4 small intestinal segments. The 4 sections were demarcated by the mid-points between the end of the duodenal loop, Meckel's diverticulum and the ileo-caecal junction. The digesta samples were freeze-dried to determine apparent digestibility of starch and CP using AIA as the inert dietary marker. Starch concentrations in diets and digesta were determined by a procedure based on dimethyl sulphoxide, α-amylase and amyloglucosidase as described by Mahasukhonthachat et al. (2010). N concentrations were determined as already stated and AIA concentrations were determined by the method of Siriwan et al. (1993). The apparent digestibility coefficients for starch and protein in 4 small intestinal sites were calculated from the following equation:

$$\text{Digestibility coefficient}=\frac{{\left(\text{Nutrient}/\text{AIA}\right)}_{\text{diet}}-{\left(\text{Nutrient}/\text{AIA}\right)}_{\text{digesta}}}{{\left(\text{Nutrient}/\text{AIA}\right)}_{\text{diet}}}$$

Starch and protein disappearance rates (g/d per bird) were deduced from the following equation:

$$\text{Nutrient disappearance rate}\left(\text{g/d per bird}\right)\;=\;F\text{eed intake}\left(\text{g/bird}\right)\text{ × Dietary nutrient}\left(\text{g/kg}\right)\text{ × Digestibility coefficient}$$

Ratios of starch to protein disappearance rates in the intestinal segments were calculated as this effectively cancels the potentially confounding influence of feed intake.

### 2.5 Statistical analyses

The experimental units were replicate cage means (6 birds per cage) and statistical procedures included model prediction and regression analysis. Response surfaces for performance parameters were generated by R Studio 1.1.456. A probability level of less than 5% was considered statistically significant. In model predictions, the non-significant coefficients were excluded which resulted in recalculation of their reduced equations for each response. When more than 1 model was significant, Akaike Information Criterion was used for model comparison and selection. Additionally, the response surface plots were constructed so that the effects from changing factors on the examined responses may be visualized. When it is relevant JMP Pro 14.0 (SAS Institute Inc. JMP Software. Cary, NC) was used to determine linear and quadratic relationships between performance parameters.

## 3 Results

### 3.1 Growth performance

The outcomes for growth performance, relative fat-pad weights and PDI values of diets are shown in Table 5. Overall growth performance averaged 2,021 g/bird for weight gain, 2,907 g/bird for feed intake and 1.439 for FCR from 14 to 35 d post–hatch. Average weight gains and FCR clearly exceeded 2019 Aviagen performance objectives for Ross 308 male broilers by 9.30% and 8.92%, respectively. The best weight gain and FCR were supported by Diet 50S50W which outperformed 2019 Aviagen objectives by 11.5% on weight gain (2,089 versus 1,849) and 12.8% on FCR (1.401 versus 1.580). Relative fat-pad weights ranged from 8.45 to 12.30 g/kg. PDI values averaged 83.6% and it was not correlated with feed intake (*r =* −0.560; *P* = 0.092). The overall mortality rate during the experimental period was 2.22%, but it was not related to treatment (*P* > 0.50).

**Table 5:** Results of growth performance, relative abdominal fat-pad weights in broiler chickens from 14 to 35 d post–hatch and pellet durability index (PDI) of experimental diets.

Diet	Weight gain, g/bird	Feed intake, g/bird	Feed conversion ratio, g/g	Fat-pad weights, g/kg	PDI, %
100S	2,021	2,903	1.437	8.45	75.0
100A	1,927	2,856	1.484	8.95	82.4
100W	1,900	2,777	1.463	12.00	95.0
50S50W	2,089	2,926	1.401	9.97	84.9
50S50A	2,001	2,946	1.473	9.86	74.0
50A50W	2,056	2,951	1.436	12.30	83.0
67S17A17W	2,038	2,936	1.411	9.45	82.4
17S67A17W	2,083	2,998	1.440	11.40	79.5
17S17A67W	2,072	2,932	1.415	10.40	92.3
33S33A33W	2,026	2,840	1.403	9.44	87.1

**Fig. 1:**
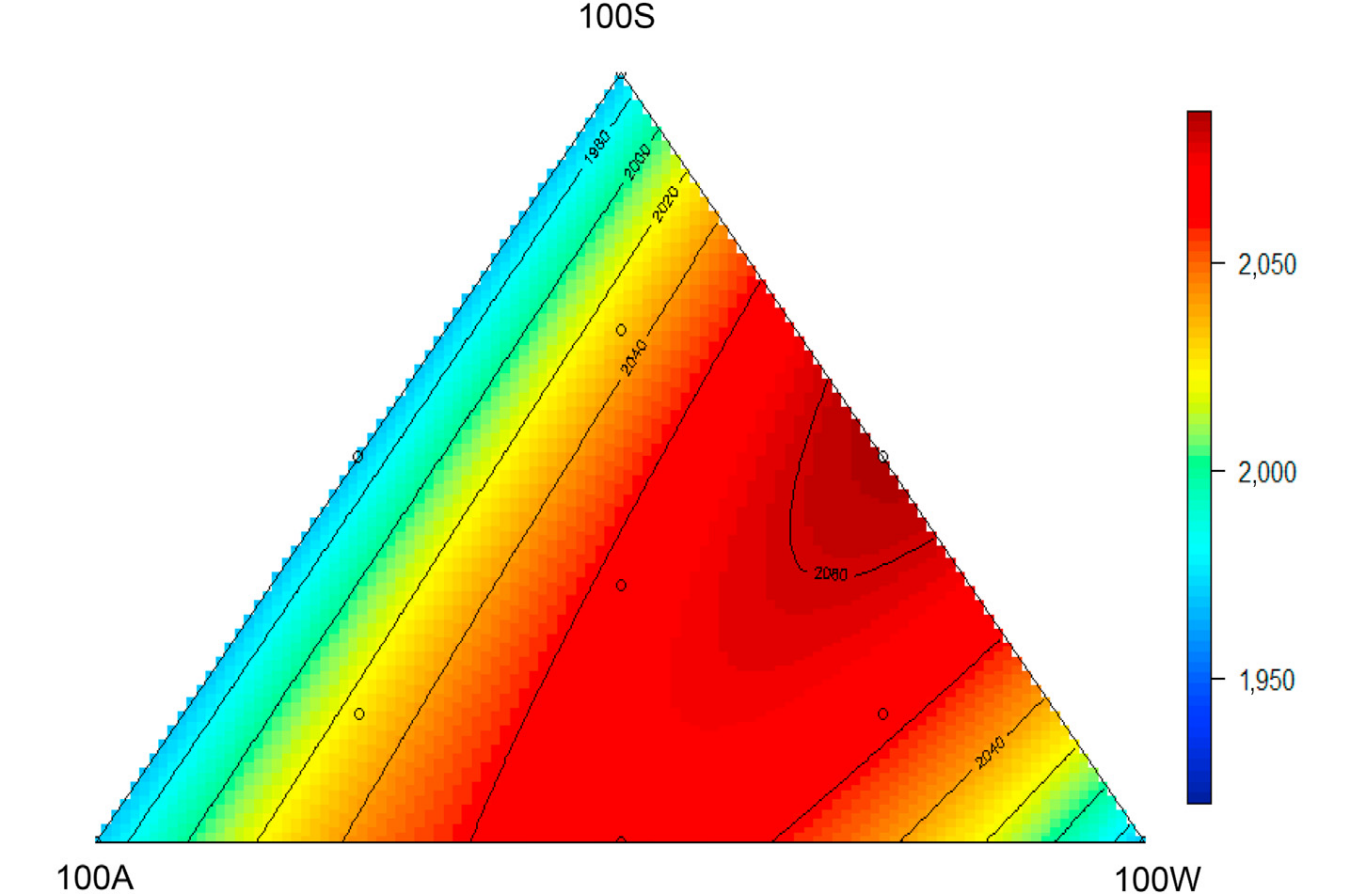
Response surface plot showing the effects of 10 dietary treatments on weight gain (g/bird, as shown in the axis) from 14 to 35 d post–hatch.

The response surface for weight gain is shown in Fig. 1, where the highest weight gain was generated by a 50:50 blend of the 100S and 100W apical diets. Weight gain can be predicted from the below equation (*R*^2^ = 0.27; *P* < 0.05):

$$\text{Weight gain}=1.968\text{×}10^{3}\left(\text{± }15.43\right)+3.992\text{×}10^{-2}\left(\text{± }0.128\right)\text{×}100\text{A×}100\text{W}+4.663\text{×}10^{-2}\left(\text{± }0.012\right)\text{×}100\text{S×}100\text{W}$$

where 100A, 100W and 100S represent the inclusion rates of Diets 100A, 100W and 100S, respectively. The error of coefficients is shown in parentheses. The same as equations below.

The relationship between feed intake and inclusions of 3 apical diets is described as below (*R*^2^ = 0.08; *P* < 0.05):

$$\text{Feed intake}=2.849\text{×}10^{3}\left(\text{± }25.85\right)+0.925\text{×}100\text{S}\left(\text{± }0.455\right)+4.426\text{×}10^{-2}\text{×}\left(\text{± }0.018\right)100\text{A×}100\text{W}$$

The predicted highest feed intake of 2,960 g/bird was generated by equal blend of 100A and 100W apical diets, which corresponds to Diet 50A50W that supported an actual feed intake of 2,951 g/kg.

**Fig. 2:**
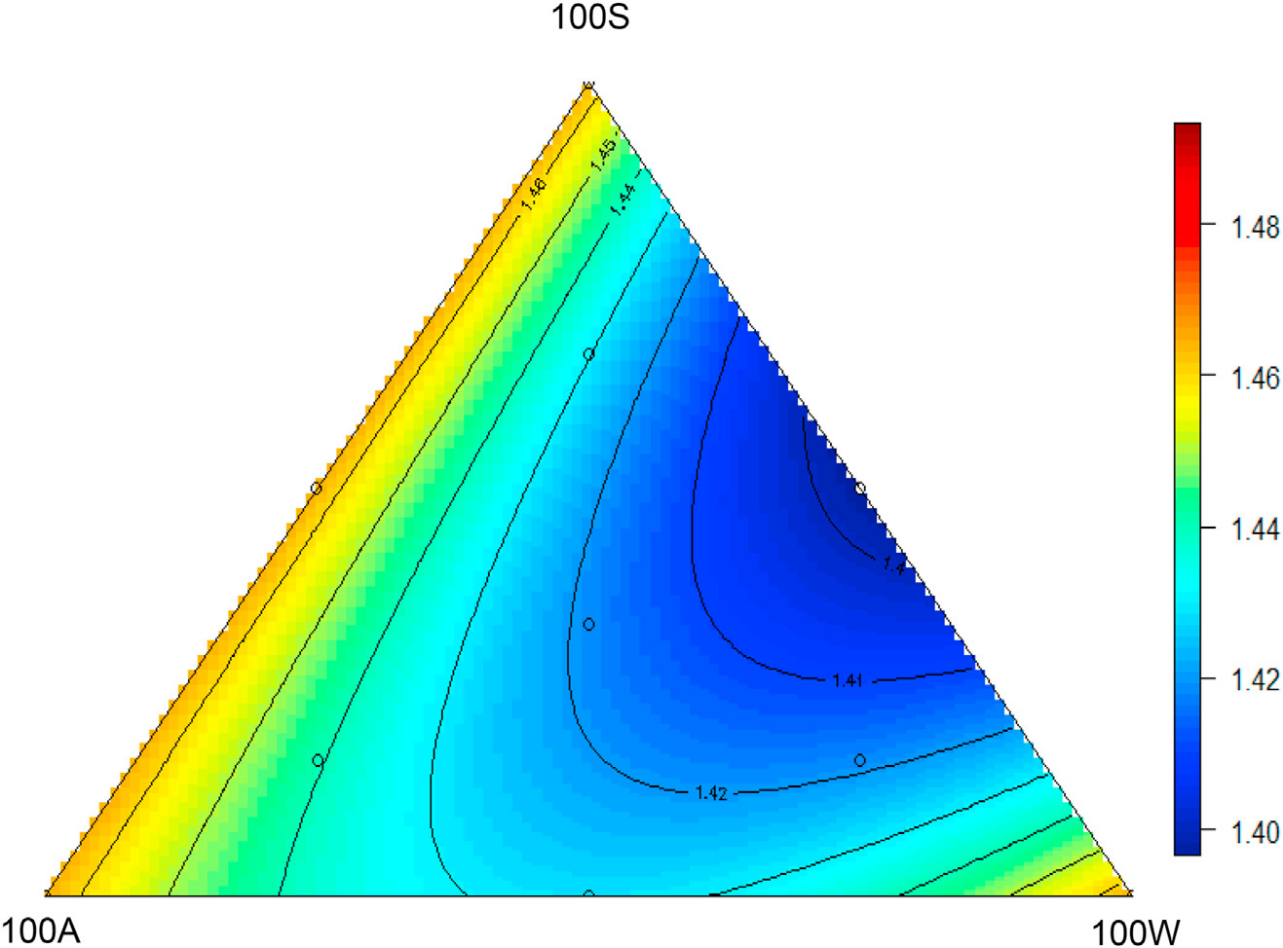
Response surface plot showing the effects of 10 dietary treatments on feed conversion ratios (g/g, as shown in the axis) of broilers from 14 to 35 d post–hatch.

The FCR response surface design is shown as Fig. 2 and the relationship (*R*^2^ = 0.29, *P* < 0.01) between the apical diets and FCR is described by the following equation:

$$\text{FCR}=\text{1.465}-1.450\text{×}10^{-5}\left(\text{± }5.99\text{×}10^{-6}\right)\text{×}100\text{A×}100\text{W}-2.695\text{×}10^{5}\left(\text{± }5.99\text{×}10^{-6}\right)100\text{S×}100\text{W}$$

The lowest predicted FCR of 1.397 in Fig. 2 is an equal blend of 100S and 100W apical diets, which corresponds to 50S50W Diet where the experimental FCR observed was 1.401.

### 3.2 Nutrient digestibility and energy utilization

**Fig. 3:**
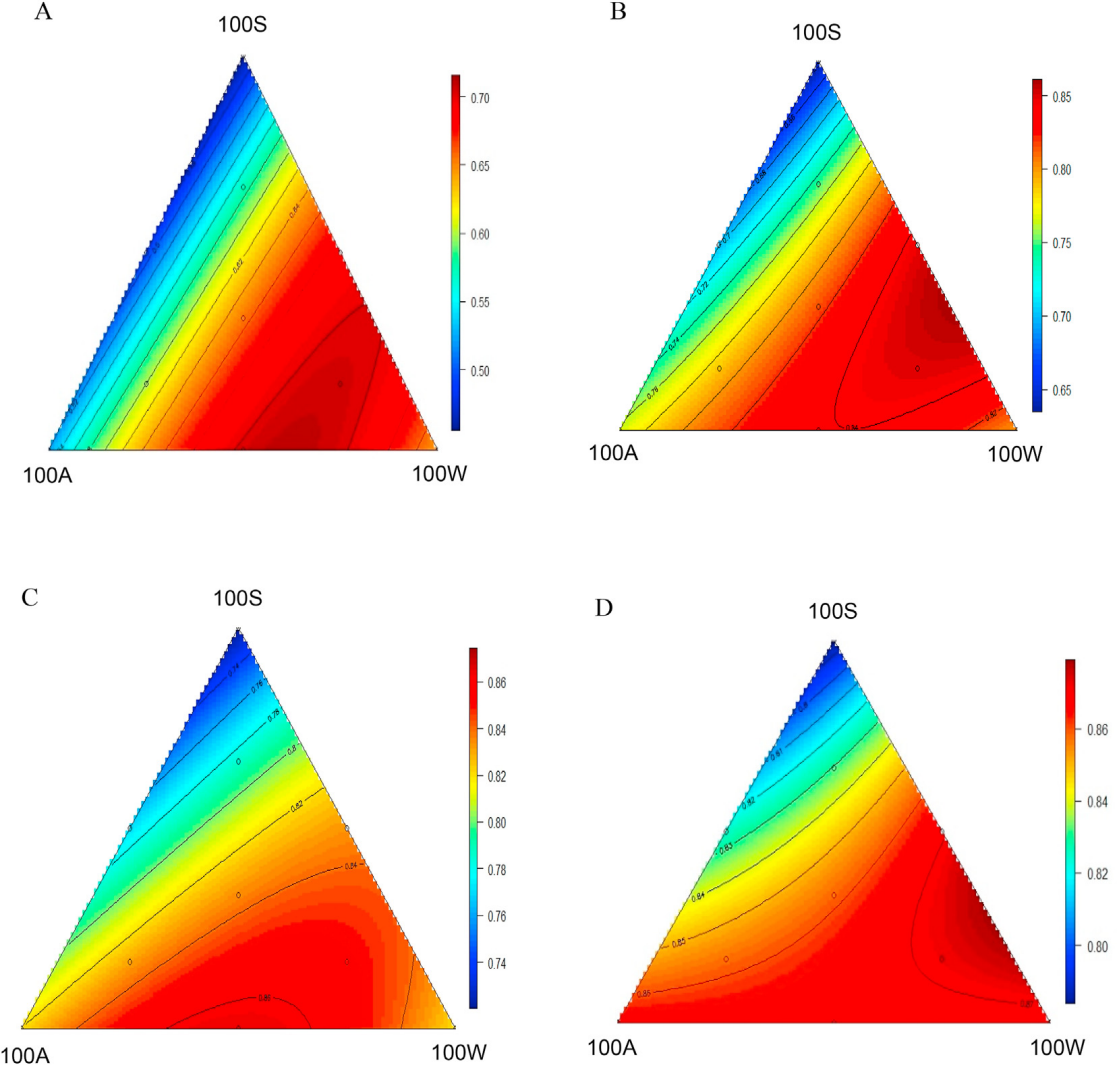
Response surface plot showing the effects of 10 dietary treatments on apparent protein digestibility coefficients in 4 small intestinal segments (A: proximal jejunum; B: distal jejunum; C: proximal ileum; D: distal ileum) of broilers as shown in each axis from 14 to 35 d post–hatch.

Apparent protein digestibility coefficients and disappearance rates in 4 small intestine segments are shown in Table 6. In addition, Fig. 3 describes responses of apparent protein digestibility at 4 sites of small intestine to change of dietary compositions. The protein digestibility in proximal jejunal (PDPJ) can be described by the following equation (*R*^2^ = 0.47, *P* < 0.001):

$$\text{PDPJ}=0.642\left(\text{±}0.038\right)+1.19\text{×}10^{-3}\left(\text{±}4.89\text{×}10^{-4}\right)\text{×}100\text{A}-1.867\text{×}10^{-3}\left(\text{±}4.92\text{×}10^{-4}\right)\text{×}100\text{S}+4.815\text{×}10^{-5}\left(\text{±}1.73\text{×}10^{-5}\right)\text{×}100\text{A×}100\text{W}+5.452\text{×}10^{-5}\left(\text{±}1.73\text{×}10^{-5}\right)\text{×}100\text{S×}100\text{W}$$

**Table 6:** Results of apparent protein (N) digestibility coefficients and disappearance rates in proximal jejunum, distal jejunum, proximal ileum, and distal ileum at 35 d post–hatch.

Diet	Apparent protein (N) digestibility coefficients				Protein disappearance rates, g/d per bird			
	Proximal jejunum	Distal jejunum	Proximal ileum	Distal ileum	Proximal jejunum	Distal jejunum	Proximal ileum	Distal ileum
100S	0.409	0.574	0.701	0.785	13.58	18.96	23.25	26.05
100A	0.481	0.726	0.785	0.853	14.56	21.95	23.72	25.79
100W	0.658	0.783	0.849	0.874	20.33	24.24	26.27	27.12
50S50W	0.685	0.823	0.819	0.866	23.72	28.53	28.36	29.98
50S50A	0.540	0.727	0.789	0.821	17.23	23.20	25.19	26.21
50A50W	0.707	0.823	0.863	0.881	21.94	25.59	26.81	27.36
67S17A17W	0.615	0.755	0.811	0.832	20.30	25.06	26.88	27.58
17S67A17W	0.623	0.769	0.817	0.839	19.68	24.31	25.81	26.50
17S17A67W	0.664	0.833	0.860	0.872	21.78	27.35	28.25	28.65
33S33A33W	0.654	0.817	0.836	0.869	20.58	25.74	26.38	27.37

The predicted highest proximal jejunal protein digestibility coefficient of 0.710 was generated by a blend of 62% 100W and 38% 100A apical diets.

Similarly, the protein digestibility in distal jejunum (PDDJ) can be explained by (*R*^2^ = 0.62, *P* < 0.001):

$$\text{PDDJ}=0.6086\left(\text{±}0.02\right)+1.701\text{×}10^{-3}\left(\text{± }2.94\text{×}10^{-4}\right)\text{×}100\text{W}+1.423\text{×}10^{-3}\left(\text{±}3.15\text{×}10^{-4}\right)\text{×}100\text{A}+2.491\text{×}10^{-3}\left(\text{±}1.04\text{×}10^{-5}\right)\text{×}100\text{A×}100\text{W}+5.738\text{×}10^{-5}\left(\text{±}1.04\text{×}10^{-5}\right)\text{×}100\text{S×}100\text{W}$$

The predicted highest protein digestibility coefficient of 0.850 was supported by a blend of 65% 100W and 35% 100S apical diets.

The equation predicting the relationship between proximal ileal protein digestibility coefficients (PDPI) is shown below (*R*^2^ = 0.43, *P* < 0.001):

$$\text{PDPI}=0.820\left(\text{±}0.012\right)-1.006\text{×}10^{-3}\left(\text{±}2.16\text{×}10^{-4}\right)\text{×}100\text{S}+2.524\text{×}10^{-5}\left(\text{±}7.76\text{×}10^{-6}\right)\text{×}100\text{S×}100\text{W}+1.792\text{×}10^{-5}\left(\text{±}8.44\text{×}10^{-6}\right)\text{×}100\text{A×}100\text{W}$$

The predicted highest protein digestibility coefficient of 0.865 stemmed from an equal blend of 100W and 100A apical diets.

The distal ileal protein digestibility coefficients (PDDI) (*R*^2^ = 0.47, *P* < 0.001) is described in following equation:

$$\text{PDDI}=0.866\left(\text{±}0.006\right)-8.274\text{×}10^{-4}\left(\text{±}1.17\text{×}10^{-4}\right)\text{×}100\text{S}+1.77\text{×}10^{-5}\left(\text{±}4.66\text{×}10^{-6}\right)\text{×}100\text{S×}100\text{W}$$

The predicted highest protein digestibility coefficient of 0.879 was supported by the blend of 73% 100W and 27% 100S diets.

The results of apparent starch digestibility coefficients and starch disappearance rates are shown in Table 7. Average starch digestibility coefficients in proximal jejunum, distal jejunum, proximal ileum, and distal ileum were 0.863, 0.926, 0.988 and 0.998, respectively. Apparent starch digestibility in proximal jejunum (0.885), distal jejunum (0.950) and proximal ileum (0.992) were correlated with percentage inclusions of three apical diets based on surface design equations where predicted optimal values are shown in parentheses. The predicted optimal starch digestibility in proximal jejunum supported by 100% of 100A diet whilst optimal values for distal jejunum and proximal ileum were supported by 50:50 blends of 100S and 100A diets.

**Table 7:** Results of apparent starch digestibility coefficients and disappearance rates in proximal jejunum, distal jejunum, proximal ileum, and distal ileum at 35 d post–hatch.

Diet	Starch digestibility coefficients				Starch disappearance rates, g/d per bird			
	Proximal jejunum	Distal jejunum	Proximal ileum	Distal ileum	Proximal jejunum	Distal jejunum	Proximal ileum	Distal ileum
100S	0.811	0.910	0.988	0.998	40.96	45.91	49.84	50.33
100A	0.869	0.928	0.984	0.998	55.35	59.06	62.63	63.57
100W	0.876	0.912	0.982	0.998	55.52	57.87	62.26	63.29
50S50W	0.851	0.930	0.991	0.998	48.86	53.42	56.92	57.36
50S50A	0.875	0.951	0.993	0.998	50.69	55.10	57.51	57.81
50A50W	0.887	0.920	0.984	0.999	60.87	63.16	67.54	68.55
67S17A17W	0.842	0.942	0.992	0.997	48.21	54.00	56.84	57.13
17S67A17W	0.876	0.920	0.990	0.999	56.47	59.85	63.76	64.32
17S17A67W	0.879	0.922	0.989	0.998	57.25	60.07	64.42	65.04
33S33A33W	0.862	0.924	0.989	0.999	48.14	51.65	55.28	55.8

**Fig. 4:**
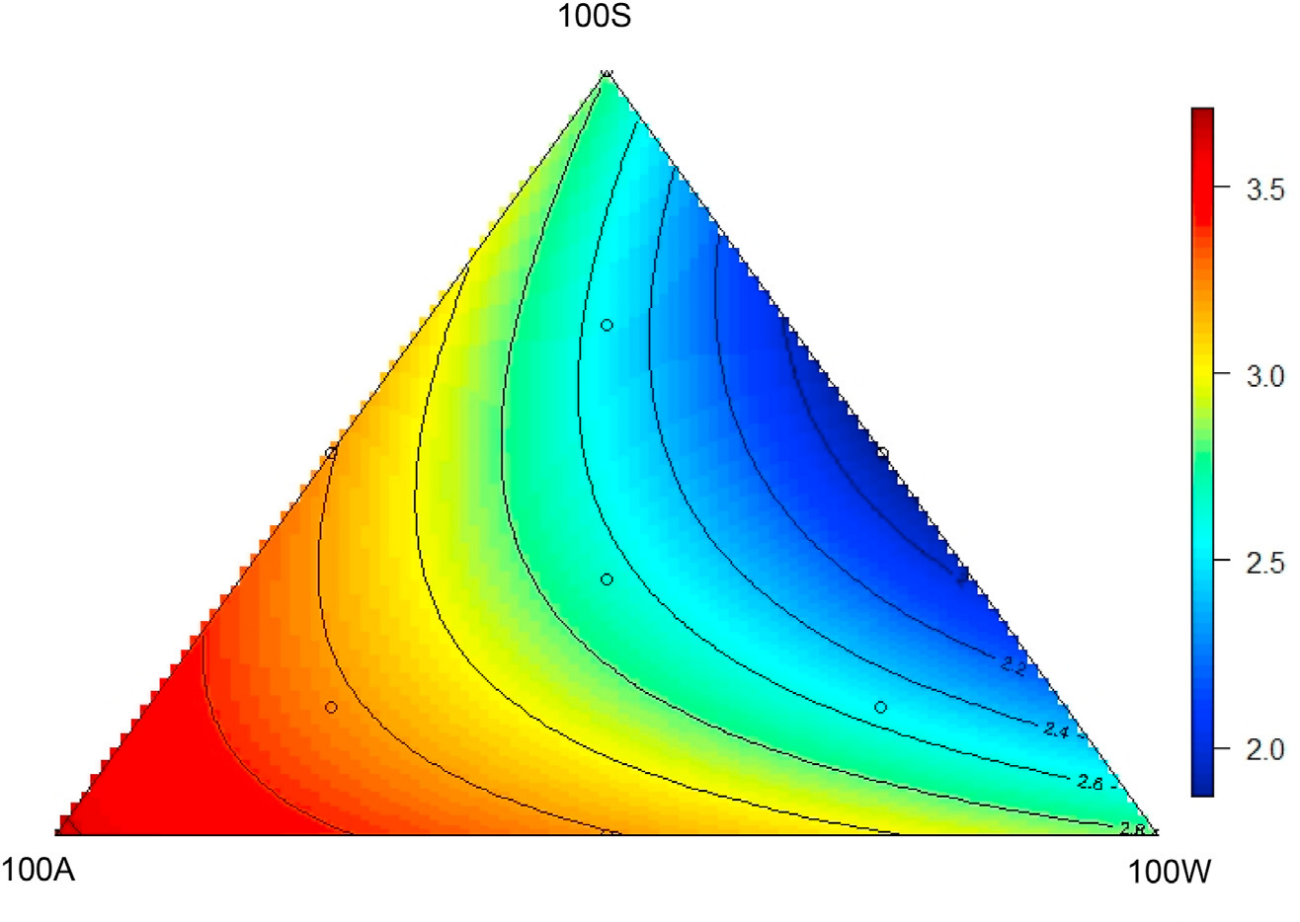
Response surface plot showing the effects of 10 dietary treatments on proximal jejunal starch:protein disappearance rate ratio as shown in the axis from 14 to 35 d post–hatch.

The results of starch:protein disappearance rate ratios are shown in Table 8. Starch:protein disappearance rate ratios were significantly correlated with FCR in all 4 small intestinal segments. The response surface design for proximal jejunal starch:protein disappearance rate ratios appear as Fig. 4. Increasing Diet100A inclusions increased starch:protein disappearance rate ratios; whereas, increasing inclusions of Diets 100S and 100W decreased ratios (*R*^2^ = 0.28, *P* = 0.001) as described by the following equation:

$$\text{Starch:protein disappearance rate ratio}=2.808\left(\text{± }0.202\right)+0.008\left(\text{± }0.004\right)\text{×}100\text{A}-3.72\text{×}10^{-4}\left(\text{±}0.0001\right)\text{×}100\text{S×}100\text{W}$$

**Table 8:** Results of starch:protein disappearance rate ratios in proximal jejunum, distal jejunum, proximal ileum and distal ileum at 35 d post–hatch and their relationship with feed conversion ratio.

Diet	Starch:protein disappearance rate ratios			
	Proximal jejunum	Distal jejunum	Proximal ileum	Distal ileum
100S	3.33	2.47	2.18	1.94
100A	4.32	2.71	2.64	2.47
100W	2.78	2.40	2.38	2.35
50S50W	2.07	1.87	2.02	1.91
50S50A	2.96	2.38	2.29	2.21
50A50W	2.78	2.48	2.52	2.51
67S17A17W	2.43	2.16	2.12	2.07
17S67A17W	2.88	2.47	2.47	2.43
17S17A67W	2.66	2.20	2.28	2.27
33S33A33W	2.28	2.00	2.07	1.82
Linear relationships with FCR				
Coefficient (*r* = )	0.464	0.512	0.313	0.372
Significance (*P* = )	<0.001	<0.001	0.014	0.003

The narrowest predicted proximal jejunal starch:protein disappearance rate ratio of 1.88 in proximal jejunum was from an equal blend of 100S and 100W diets.

The effects of dietary treatments on nutrient utilisation in broiler chickens are shown in Table 9. The response in ME:GE ratios can be described by the following equation:

$$\text{ME:GE ratio}=0.821\left(\text{±}0.0043\right)+4.427\text{×}10^{-4}\left(\text{±}0.0001\right)\text{×}100\text{W}-2.609\left(\text{±}0.0001\right)\text{×}100\text{S}$$

**Table 9:** Results of nutrient utilisation in broiler chicken from 27 to 29 d post–hatch.

Diet	AME, MJ/kg DM	ME:GE ratio	N retention, %	AMEn, MJ/kg DM
100S	15.31	0.80	71.16	13.45
100A	15.14	0.82	70.98	13.51
100W	16.18	0.87	74.04	14.31
50S50W	15.42	0.83	71.98	13.42
50S50A	14.98	0.80	70.85	13.16
50A50W	15.59	0.85	71.65	13.83
67S17A17W	15.48	0.81	71.14	13.58
17S67A17W	15.42	0.83	74.18	13.58
17S17A67W	15.69	0.84	71.61	13.80
33S33A33W	15.62	0.83	70.71	13.89

AME = apparent metabolizable energy; ME:GE ratio = metabolizable to gross energy ratio, N = nitrogen; AMEn = N-corrected AME.

The predicted optimal ME:GE ratio of 0.87 was supported by the 100W diet.

## 4 Discussion

In order to formulate iso-energetic diets, dietary starch and fat concentrations were varied in experimental diets. However, there were no correlations between growth performance and calculated concentrations of any nutrients including dietary starch and fat. Analysed total amino acid concentrations in the 3 apical diets were shown in Table 4. Amino acid concentrations of the remaining 7 blended diets were calculated and there was no correlation between growth performance and dietary amino acid concentrations (*P* > 0.05). Interestingly, abdominal fat pat weights were positively correlated with analysed dietary starch concentrations (*r =* 0.748, *P* = 0.013). Moreover, energy utilisation expressed as ME:GE ratios were also positively correlated with analysed dietary starch concentrations (*r =* 0.786, *P* = 0.007). Animal's ability of converting surplus carbohydrate into fat in adipose tissue was known for decades (Flatt, 1970). In the present study, the transition from Diet 100S to Diet 100A reduced soybean meal inclusion from 323 to 162 g/kg and increased wheat inclusion from 577 to 689 g/kg. Consequently, dietary starch increased from 325 to 415 g/kg when dietary CP reduced from 214 to 197 g/kg. It remains a challenge to minimise carcass fat when developing reduced CP diets as the least-cost feed formulation prefers grain rather than added oil as the primary energy source in reduced CP diets to avoid high inclusions of filler. This led to the difficulty of investigating the benefit of capping dietary starch to protein ratios in reduced CP diets (Greenhalgh et al., 2020).

The concept of true protein was experimented when formulating diets for the present experiment. CP has been used for over 150 years and is simply the nitrogen content of the feed multiplied by 6.25. It assumes that, on average, feed ingredients contain 160 g/kg protein (Jones, 1941). As a measure of the nitrogen content of feed, the use of CP may be justified in practical diet formulation since most commercial broiler diets are formulated to digestible amino acid although a number of research data make use of total amino acid. True protein, as reflected by the total of notionally “essential amino acids” and “non-essential amino acids” may hold more relevance in diets containing high level of NBAA. Grau (1948) first proposed that the lysine requirements of chicks was a function of the CP content of the diet over a range of 50 to 300 g/kg. This early work suggested that lysine requirement for maximum growth at a particular CP level increases as dietary CP increases. Alhotan and Pesti (2016) remodelled digestible lysine requirment as a proportion of true protein and they endorsed true protein is a better indicator than CP for digestible lysine requirment. Moreover, research utilised diets with high level of supplemental amino acid doesn't define what values of CP have been used for the supplemental amino acid, whilst several researchers do not assign CP values to the supplemental amino acid (Aftab et al., 2006). Therefore, experimental diets in the present study were formulated to contain the same true protein content (203 g/kg) where 1 g of NBAA equals to 1 g of true protein.

Previously, similar experiments showed that diets containing highest soybean meal and lowest NBAA often led to the best growth performance (Chrystal et al., 2020a, Chrystal et al., 2020b; Greenhalgh et al., 2020). In contrast, in the present study, the best weight gain and lowest FCR was predicted in broiler chickens offered an equal blend of 100S and 100W diets which is respective to Diet 50S50W. Apparent digestibility of starch and protein were determined at 4 sites of small intestine to explore the relevance of digestion rate with growth performance especially FCR. FCR was not correlated with apparent distal ileal digestibility coefficient (*P* = 0.560) of protein but was correlated with apparent protein digestibility in proximal jejunum (*r =* −0.369, *P* = 0.040) and distal jejunum (*r =* −0.316, *P* = 0.015). In Diet 50S50W, protein was derived from soybean meal (35.7%), wheat (30.5%), whey protein concentrate (27.7%) and NBAA (6.03%), where the approximate percentages are shown in parentheses. The unequal contributions to dietary CP from different protein source may have resulted different rate of protein digestion as reflected in jejunal protein digestibility. Pancreatic secretions of amylase (and trypsin) are found in highest concentrations in the jejunum and enzyme activity in the small intestine decreases both proximally and distally (Bird, 1971). Indeed, the present study suggested average 89.8% of the total digestible protein had been digested by the end of jejunum. Similar correlations between FCR and jejunal protein digestibility were reported in sorghum-based diets (Liu and Selle, 2015). In the present study, mean starch and protein disappearance rate ratios were linearly related to both weight gain (*r =* −0.277; *P* = 0.031) and FCR (*r =* 0.502; *P* < 0.001), which suggested narrower starch and protein disappearance rate ratios are preferred for better feed conversion and weight gain. It is also curious that broiler chicken offered Diet 100A did not generate the highest jejunal digestibility as Diet 100A contained the highest level of NBAA and NBAA is considered to be rapidly available following ingestion (Fig. 4). The whey protein concentrate is readily digestible in broiler chickens was clearly demonstrated by Chrystal et al., 2020a, Chrystal et al., 2020b. In that study, 195 g/kg soybean meal was replaced with 124 g/kg whey protein in maize-based diets with 165 g/kg CP contents. Consequently, the average digestibility coefficient of 17 amino acids was numerically increased by 7.41% (0.638 versus 0.594; *P* = 0.251) in distal jejunum and significantly increased by 7.85% (0.852 versus 0.790; *P* = 0.007) in distal ileum. The 100S apical diet may have been disadvantaged by anti-nutritive factors inherent in soy including trypsin inhibitor, phytate, tannins and oligosaccharides; trypsin inhibitor has been shown to compromise amino acid digestibility (Clarke and Wiseman, 2007) and phytate has been shown to exacerbate flow of endogenous amino acids to the intestine in poultry (Onyango et al., 2008). The various constituents in feedstuffs have been considered by Ravindran (2016). NBAA are notionally 100% digestible (Lemme et al., 2005); nevertheless, birds offered the 100W apical diet digested protein more rapidly. One possible interpretation, is that intact whey protein is readily converted to di- and tri-peptides and these oligopeptides are absorbed more rapidly and effectively than NBAA.

It may be deduced from Table 6, Table 7 that 99.8% of starch was digested along the small intestine overall and 92.6% was digested in the jejunum with little variation across dietary treatments. In contrast, 84.9% of dietary protein was digested along the small intestine and 76.3% was digested in the jejunum with substantial variation between diets. Essentially, the Na^+^-dependent transport system, SGLT-1, drives intestinal uptakes of glucose (Röder et al., 2014) and endogenous flows of glucose are negligible. Alternatively, intestinal uptakes of monomeric (or single) amino acids are driven by an array of Na^+^-dependent and Na^+^-independent transporters with overlapping specificities (Hyde et al., 2003). However, di- and tri-peptides are principally absorbed via the oligopeptide transporter, PepT-1 (Zwarycz and Wong, 2013). Moreover, the likelihood is that majority of amino acids are absorbed as oligopeptides (Matthews, 1983) and oligopeptides are absorbed more rapidly and efficiently than single or NBAA (Daniel, 2004; Gilbert et al., 2008).

The molecular weight distribution of peptides in wheat, soybean meal and whey protein were determined by size-exclusion chromatography as shown in Table 10. It is reasonable to classify peptides with molecular weights of less than 500 Da as oligopeptides as the average molecular weight of amino acids is 136 Da. Soybean meal contained 23.3% oligopeptides, wheat 11.0%, but whey protein contained less than 1% oligopeptides. Whey protein concentrate predominantly contains large polypeptides (Jeewanthi et al., 2017), which is consistent with 97.3% of whey protein peptides exceeding 10,000 Da in the present study. Szczurek et al. (2013) offered diets containing 0, 8 and 32 g/kg WPC (804 g/kg CP) to broiler chickens to 42 d post–hatch. These inclusions improved FCR by 6.77% and 11.5% and, 32 g/kg WPC increased ileal protein digestibility by 7.31% (0.851 versus 0.793) at 26 d post–hatch. The 100W apical diet supported more rapid proximal jejunal protein disappearance rates than the 100S and 100A apical diets by 49.7% and 39.6%, respectively, in the present study (Table 6). This may suggest that polypeptides in whey are readily converted to oligopeptides in the avian gut, which facilitates intestinal uptakes of amino acids as oligopeptides.

**Table 10:** Peptide distribution in feedstuffs determined by peptide size-exclusion chromatography.

Molecular weight distribution, Da	Feedstuff, %		
	Wheat	Soybean meal	Whey
>10,000	65.5	47.4	97.3
10,000 to 2,000	11.0	4.8	1.5
2,000 to 500	12.5	24.5	0.4
<500	11.0	23.3	0.8

## 5 Conclusion

The present study explored the relevance of protein-bound and non-bound amino acid digestive dynamics on growth performance and nutrient utilisation in broiler chickens. FCR was correlated with distal jejunal digestibility coefficients of protein but not with apparent ileal protein digestibility. It is concluded that rate of protein digestion needs to be considered in diets with reduced SBM and increased NBAA inclusions.

## Author contributions

**S. Y. Liu** was the principal investigator of the relevant project and is the corresponding author. All co-authors were variously involved in completion of this paper. **S. P. Macelline** conducted and supervised the feeding study. **P.V. Chrystal** formulated the diets. **P. H. Selle** and **S. P. Macelline** completed the statistical analyses. All authors contributed to writing and editing of the manuscript.

## Declaration of competing interest

We declare that we have no financial and personal relationships with other people or organizations that can inappropriately influence our work, and there is no professional or other personal interest of any nature or kind in any product, service and/or company that could be construed as influencing the content of this paper.
